# Testing a Novel Web-Based Neurocognitive Battery in the General Community: Validation and Usability Study

**DOI:** 10.2196/25082

**Published:** 2021-05-06

**Authors:** Riley Capizzi, Melissa Fisher, Bruno Biagianti, Neelufaer Ghiasi, Ariel Currie, Karrie Fitzpatrick, Nicholas Albertini, Sophia Vinogradov

**Affiliations:** 1 Department of Psychology Temple University Philadelphia, PA United States; 2 Department of Psychiatry and Behavioral Sciences University of Minnesota Minneapolis, MN United States; 3 Department of Research and Development Posit Science Corporation San Francisco, CA United States

**Keywords:** cognition, normative, remote, digital, online, web-based, BrainHQ, Posit Science Corporation

## Abstract

**Background:**

In recent years, there has been increased interest in the development of remote psychological assessments. These platforms increase accessibility and allow clinicians to monitor important health metrics, thereby informing patient-centered treatment.

**Objective:**

In this study, we report the properties and usability of a new web-based neurocognitive assessment battery and present a normative data set for future use.

**Methods:**

A total of 781 participants completed a portion of 8 tasks that captured performance in auditory processing, visual-spatial working memory, visual-spatial learning, cognitive flexibility, and emotional processing. A subset of individuals (n=195) completed a 5-question survey measuring the acceptability of the tasks.

**Results:**

Between 252 and 426 participants completed each task. Younger individuals outperformed their older counterparts in 6 of the 8 tasks. Therefore, central tendency data metrics were presented using 7 different age bins. The broad majority of participants found the tasks interesting and enjoyable and endorsed some interest in playing them at home. Only 1 of 195 individuals endorsed *not at all* for the statement, “I understood the instructions.” Older individuals were less likely to understand the instructions; however, 72% (49/68) of individuals over the age of 60 years still felt that they *mostly* or *very much* understood the instructions.

**Conclusions:**

Overall, the tasks were found to be widely acceptable to the participants. The use of web-based neurocognitive tasks such as these may increase the ability to deploy precise data-informed interventions to a wider population.

## Introduction

### Background

For decades, neuropsychological methods have been leveraged to understand and characterize the relative strengths and weaknesses of individuals experiencing an array of neuropsychiatric syndromes [1-5]. These profiles have also been shown to predict future deterioration in Alzheimer disease and other cognitive and functional outcomes in schizophrenia, bipolar disorder, depression, and traumatic brain injury [6-8]. However, traditional paper and pencil assessments are lengthy and expensive and require considerable training on the part of the assessor. Given these concerns and the rising availability of personal mobile technologies, a movement to capture reliable cognitive functioning digitally has begun [9-12]. Although remote cognitive technologies are still budding, they promise several benefits in clinical and research settings.

Remote platforms greatly increase the accessibility of psychological testing. The National Institute of Mental Health has established a priority to reach typically underserved populations, such as individuals living with limited physical mobility or in rural areas [13]. Digital measures allow testing to be conducted within the comfort of one’s own home, thereby increasing the ability to serve historically difficult-to-reach patients. Remote testing would also likely reduce the time providers spend scheduling and carrying out in-person assessments. These benefits may also help to reduce assessment costs. Accessibility concerns have taken center stage during the current global pandemic, highlighting the need for reliable, easy-to-use, and remote measures.

In addition to opportunities for accessibility, digital remote methods for cognitive testing encourage the use of longitudinal assessments. Repeated measurements provide a wealth of information above and beyond individual snapshots of performance [14]. A study of individuals at clinical high risk for conversion into a psychotic disorder found that although baseline measures of cognition helped differentiate at-risk individuals from healthy participants, cognitive trajectories followed for 2 years differentiated converters from nonconverters [15]. Another study of healthy older adults found that repeated memory assessments pushed to a handheld device were widely accepted and showed associations with the hippocampal brain structure, whereas typical baseline measures of cognition did not [16].

Although in-person neuropsychological methods unquestionably have their benefits and function optimally in distraction-free testing environments, the generalizability of data collected in these unique settings has been questioned [17]. There is a growing body of literature evaluating assessments that can be administered in people’s daily lives, thus potentially capturing more meaningful data on how they function within their own environments [18,19]. For example, a clinician may be more interested in how their patient performs cognitively while completing their night shift at work rather than in a highly controlled testing center at midday.

Digital methods also provide a means to measure important metrics such as reaction time (RT) and trial-by-trial performance, which have also shown associations with clinical outcomes [20-22]. These potential benefits, alongside the added ability of electronic health record integration, may further facilitate person-centered practice and move us closer toward the goal of precision medicine [23]. However, little to no normative data currently exists for these task batteries, limiting the interpretability of new findings [24].

A particular novel mobile assessment platform implemented by the Posit Science Corporation has shown recent utility capturing cognitive performance differences common to psychiatric disorders, such as schizophrenia and depression, using a neuroscience-informed approach to task development [25,26]. These tasks were designed to capture the variability associated with aberrant neural mechanisms underlying an individual’s ability to flexibly acquire new information and adapt to changing cognitive and emotional demands, integral processes for normative cognitive functioning and general learning ability [26-29]. Sound sweeps, a task of auditory processing speed, showed improvements during a cognitive training intervention in individuals with recent-onset and chronic schizophrenia [30]. The greatest improvement was observed during the first 20 hours of training, followed by a plateau at subsequent assessment points. Intraindividual variability in the time taken to reach this plateau was also associated with the likelihood that the intervention would generalize to other untrained cognitive domains, suggesting a potential target-mediated treatment response. Another study found that improvements in sound sweeps performance after cognitive training were correlated with gains in working memory and global cognition [31]. Despite the apparent utility of these tasks, they have yet to be adequately evaluated in normative data samples.

### Objectives

Therefore, we set out to test a battery of 8 web-based cognitive tasks developed by Posit Science in samples drawn from the general community. Sensory perception, social cognition, and executive functions were digitally assessed in-person with state fair attendees and remotely through testing of college students. In addition, the usability and feasibility of this new battery were investigated in a subset of participants.

## Methods

### Participants

All study procedures were approved by the institutional review board of the University of Minnesota (UMN). Recruitment and study participation took place either in-person at the Minnesota State Fair (MSF) or remotely for course credit at UMN. The MSF is the second-most highly attended state fair in the United States, with approximately 2 million Midwesterners visiting every year [32]. State fairgoers represent a wide array of demographic backgrounds [33]. MSF participants were asked to participate if they were aged between 18 years and 80 years (inclusive), and UMN students were asked to participate if they were aged between 18 years and 40 years (inclusive). Inclusion criteria for all participants were (1) no visual, auditory, or motor impairments that would prevent completion of the assessments; (2) fluent and literate in English; and (3) no use of illicit substances or alcohol over the prior 8 hours. We received cognitive data from 816 participants and usability survey responses from 219 participants who participated in a substudy. However, cognitive data from 35 participants who took the battery multiple times were omitted from the primary cognitive analyses below, and survey data were omitted for another 24 participants who completed usability questionnaires multiple times. Therefore, 781 participants were included in the primary analyses, and survey data from 195 participants were inspected (Table 1).

**Table 1:** Participant demographics by sample.

Demographics			Sample				Total (N=781)
			College (n=202)		State fair (n=579)		
**Gender^a^**							
	Female, n (%)	144 (71.6)		338 (58.6)		482 (62.0)	
	Male, n (%)	57 (28.4)		237 (41.1)		294 (37.8)	
	Intersex, n (%)	0 (0.0)		2 (0.3)		2 (0.3)	
Age, mean (SD)			20.12 (2.28)		44.82 (17.72)		38.4 (18.7)
Years of education, mean (SD)			14.59 (1.5)		16.57 (2.77)		16.1 (2.65)
Grade point average, mean (SD)^b^			3.45 (0.35)		N/A^c^		3.45 (0.35)
Occupation level, mean (SD)^d^			5.16 (1.68)		3.06 (1.84)		3.81 (2.05)

^a^Three participants preferred not to respond.

^b^Grade point average only collected in the undergraduate samples.

^c^N/A: not applicable.

^d^Hollingshead two-factor index: occupational scale.

### Procedures

After receiving informed consent, the study participants provided demographic information and completed a battery of cognitive tasks. College students participated remotely via their own personal devices (eg, tablets or personal computers) and were asked to find a stable internet connection to complete the tasks in a quiet, private environment using headphones, preferably over-the-ear headphones. MSF participants completed the study procedures in an enclosed fair structure using lab iPads (Apple Inc) and over-the-ear headphones. Participants were randomly assigned to complete 3 to 4 of the 8 cognitive assessments. State fair participants completed a subset of the measures because of the time limit set by the Driven to Discover State Fair program. A maximum of 30 minutes was allowed for giving consent and the completion of demographic information, cognitive measures, and questionnaires. Participants were randomly assigned to complete 1 of 2 cognitive batteries that were equivalent in length and comparable in terms of the number of auditory, visual, and social cognition tests.

### Online Neurocognitive Assessments

Our work aims to translate measures from basic cognitive neuroscience into short, computerized assessments of discrete cognitive processes that individuals can easily complete with minimal assistance in various settings [25]. These assessments are designed to enable the interpretation of specific deficits that could signal that an individual is experiencing cognitive difficulties and impaired learning ability.

The first step in the development of the assessment suite was to decide on the cognitive domains and processes that are known to play a critical role in an individual’s ability to learn new information, to interact adaptively with cognitive and emotional challenges in the environment, and to adapt to new learning demands. In line with the principles of team science, we integrated theoretical perspectives, technical expertise, and empirical knowledge drawn from a team of cognitive neuroscientists working in human and animal model systems, clinical researchers, and preclinical translational behavioral neuroscientists. Through a consensus-building process, we identified 3 critical neural domains: (1) perceptual processing (sound sweeps and beep seeker), (2) executive functioning (bubble pop, pathfinder, and mind bender), and (3) social-emotional processing (face to face, tap the emotion, and emotional face). Within each domain, we identified constructs, that is, a definable cognitive process that could be measured at the behavioral level and for which there existed clearly hypothesized and measurable neural-circuit mechanisms (eg, for executive functioning, set-shifting). We identified a cognitive neuroscience paradigm that could selectively and parametrically measure each of these constructs at the behavioral level. Guided by item response theory, these assessments use adaptive testing models to adjust the difficulty level according to the user, thereby reducing the duration of the test [34]. Online neurocognitive assessments (ONAs) take an average of 184 (SD 201) seconds to complete. Instructions on sampling the ONAs are presented in Multimedia Appendix 1.

#### Beep Seeker: Auditory Discrimination and Sensory Memory

Beep seeker is an auditory discrimination task in which participants are presented with a target tone and are asked to identify it in later trials amidst 2 other distractor tones. Accurate identification of the target tone prompts subsequent trials with more similar distractor tones. A linear staircase method was used to identify the participant’s discrimination threshold on a scale of 1 to 15, with higher scores indicating better performance.

#### Sound Sweeps: Auditory Perception and Processing Speed

Sound sweeps is an auditory perception task in which participants are presented with 2 consecutive tones that may either sweep from a low to high pitch or high to low pitch. The participants were asked to identify the direction of each sweep. The sweep sounds’ speed varied according to trial performance, and participant performance was measured as log_10_(average RT in seconds).

#### Bubble Pop: Visual-Spatial Working Memory

Bubble pop is a working memory task in which participants are asked to follow a set of target bubbles that independently move around the screen alongside other distractor bubbles. The number of target bubbles varied with the performance. Accuracy was measured as the number of bubbles correctly tracked using a 2-up, 1-down staircase method.

#### Pathfinder: Visual-Spatial Learning

Pathfinder is a learning test. A path with 15 nodes is presented. The participant then attempted to recreate the path from memory. If a node is missed, the trial ends, and the path is shown again (5 trials in total). Accuracy was measured as the percentage of nodes correctly recalled out of the total number of nodes.

#### Mind Bender: Cognitive Flexibility

Mind bender is a task that instructs participants to identify images that follow an established rule around other pictures that violate the rule. Performance was measured as log_10_(correct trial RT in milliseconds).

#### Tap the Emotion: Emotion Detection and Inhibitory Control

Tap the emotion requires the participant to tap the screen when a happy or sad face appears and inhibits the prepotent response to tap when presented with a neutral face. Performance was measured as the mean accuracy of neutral trials.

#### Face to Face: Emotion Identification

A face is shown with a specific emotion, followed by a series of faces with various emotions. Participants were asked to identify the target emotion first shown among the series of faces. The task varies in difficulty by changing the number of emotions presented and the number of faces to choose from. Performance was measured as log_10_(duration of target stimulus presentation).

#### Emotional Face: Inhibitory Control of Emotion

Emotional face provides a measure of executive attention, in which an expressive face is shown and overlaid with a congruent or incongruent word (Stroop effect). The increased RT to incongruent stimulus combinations captures the capacity for conflict resolution. Performance was measured by subtracting the mean RT of the correct congruent trials from the mean RT of the correct incongruent trials.

### Usability Questionnaire

This lab-designed measure asked participants to indicate how much they agreed with a series of 5 statements using a 5-point Likert scale ranging from *not at all* to *very much*. The specific questions are shown in Multimedia Appendix 2.

### Statistical Analysis

All main outcomes were screened for normality. Although some measures showed skewness and kurtosis (eg, beep seeker, tap the emotion, and face to face), all had adequate variance. The task distributions are shown in Multimedia Appendix 1. Nonparametric statistics were subsequently used for beep seeker, tap the emotion, and face to face. Outliers greater than 2.5 SD from the mean were winsorized and represented less than 1% of the data. For ease of comparison, outputs from sound sweeps, mind bender, and face to face were transformed so that higher scores indicated better performance.

General tendencies, dispersion metrics, and associations with demographic variables were calculated for each ONA. A total of 2 individuals identified as intersex were excluded from the gender comparison of ONA performance because of insufficient power. Given that 6 of 8 ONAs showed significant associations with age, task performance was also summarized across 7 different age ranges. To investigate concurrent and discriminant validity across ONAs, a Spearman rank-order correlation matrix was summarized; however, a factor analysis was not conducted because of inefficient overlap of measures completed across samples and a subsequent lack of power.

## Results

In total, 781 individuals completed at least one ONA (college, n=202; MSF, n=579). Table 2 presents the means, SD, and ranges for each task.

**Table 2:** Normative statistics by online neurocognitive assessment.

Assessments	Participant, n (%)	Task metric, mean (SD)	Minimum^a^	Maximum^b^
Beep seeker	269 (34.4)	5.01 (4.63)	1.00	15.00
Sound sweeps^c^	269 (34.4)	0.98 (0.33)	0.00	1.70
Bubble pop	293 (37.5)	5.07 (1.20)	1.00	7.80
Path finder	323 (41.4)	52.32 (24.97)	1.00	100.00
Mind bender^c^	256 (32.8)	−9.09 (1.03)	−11.60	−6.80
Tap the emotion	426 (54.5)	78.48 (17.88)	20.00	100.00
Face to face^c^	339 (43.4)	−2.03 (0.42)	−3.45	−1.50
Emotional face	252 (32.3)	53.19 (80.11)	−169.00	342.90

^a^Minimum value refers to the lowest task metric across participants on a given task.

^b^Maximum value refers to the highest task metric across participants on a given task.

^c^Raw task output was inverted so that higher scores indicated better performance.

Over 96.4% (188/195) of participants found the ONAs to be at least *a little bit* interesting enjoyable. Almost 91.8% (179/195) of individuals found the tasks at least *a little bit* easy. Only 1 participant endorsed *not at all* to the statement, “I understood the instructions.” The vast majority of participants found the ONA instructions easy to understand at the level *of a little bit* or more. Age was found to be negatively associated with the extent to which people agreed that they understood the instructions (ρ=−0.21; *P*=.004). Still, over 72% (49/68) of participants over the age of 60 years reported that they *mostly* or *very much* aligned with the statement, “I understood the instructions.” In addition, when asked how much people agreed with the statement, “I would play these games at home,” 69.2% (135/195) endorsed *a little bit* or more.

Task relationships with demographic variables such as gender, age, and education are presented in Table 3 and those with occupation level and grade point average (GPA) are presented in Table 4. Male participants outperformed female participants on beep seeker (Wilcoxon rank-sum [W]=6927; *P*=.01), sound sweeps (two-tailed *t* test: *t*_163.65_=−2.12; *P*=.04), and path finder (two-tailed *t* test: *t*_264.93_=−4.11; *P*<.001). Age was negatively correlated with task performance on sound sweeps (Pearson correlation [*r*]=−0.33; *P*<.001), bubble pop (*r*=−0.50; *P*<.001), path finder (*r*=−0.38; *P*<.001), mind bender (*r*=−0.41; *P*<.001), tap emotion (ρ=−0.24; *P*<.001), and face to face (*r*=−0.19; *P*<.001). Given the consistent association with age, task performance is displayed across 7 age bins, as shown in Tables 5 and 6. Counterintuitively, fewer years of education were associated with better performance on bubble pop (*r*=−0.13; *P*=.03), mind bender (*r*=−0.13; *P*=.04), and tap the emotion (ρ=−0.16; *P*=.001); however, when controlling for age, these relationships were not significant. Higher levels of occupation were significantly associated with an elevation in beep seeker (ρ=0.15; *P*=.02). Lower levels were associated with better performance on sound sweeps (*r*=−0.16; *P*=.03), bubble pop (*r*=−0.17; *P*=.01), path finder (*r*=−0.24; *P*<.001), mind bender (*r*=−0.21; *P*=.001), and tap the emotion (ρ=−0.20; *P*<.001), but when age was accounted for, only bubble pop remained associated (*F_1,227_*=4.12; *P*=.04). GPA was not significantly correlated with any of the ONAs.

**Table 3:** Association of online neurocognitive assessments with demographic variables (gender, age, and education).

Assessments	Demographics																		
	Gender							Age (years)							Education (years)				
	Student two-tailed *t* test			Wilcoxon ranked sum			Pearson correlation				Spearman rank-order correlation			Pearson correlation				Spearman rank-order correlation	
	*t* test (*df*)	*P* value	W		*P* value	*r*			*P* value	ρ		*P* value	*r*			*P* value	ρ		*P* value
Beep seeker	—^a^	—	6927		.01	—			—	0.10		.11	—			—	0.10		.11
Sound sweeps^b^	−2.12 (163.65)	.04	—		—	−0.33			<.001	—		—	<0.001			.99	—		—
Bubble pop	−1.09 (185.61)	.28	—		—	−0.50			<.001	—		—	−0.13			.03	—		—
Path finder	−4.11 (264.93)	<.001	—		—	−0.38			<.001	—		—	−0.10			.07	—		—
Mind bender^b^	−1.39 (145.12)	.17	—		—	−0.41			<.001	—		—	−0.13			.04	—		—
Tap the emotion	—	—	19,480		.28	—			—	−0.24		<.001	—			—	−0.16		.001
Face to face^b^	—	—	12,984		.85	—			—	−0.19		<.001	—			—	−0.01		.85
Emotional face	1.70 (223.3)	.09	—		—	−0.03			.61	—		—	0.01			.82	—		—

^a^Nonparametric test provided given violation of normality.

^b^Raw task output was inverted so a larger output indicated better performance.

**Table 4:** Association of online neurocognitive assessments with demographic variables (occupation level and grade point average).

Assessments	Demographics								
	Occupation level					Grade point average			
	Pearson correlation		Spearman rank-order correlation		Pearson correlation			Spearman rank-order correlation	
	*r*	*P* value	ρ	*P* value	*r*		*P* value	ρ	*P* value
Beep seeker	—^a^	—	0.15	.02	—		—	0.07	.57
Sound sweeps^b^	−0.16	.03	—	—	0.08		.39	—	—
Bubble pop	−0.17	.01	—	—	0.04		.67	—	—
Path finder	−0.24	<.001	—	—	0.13		.26	—	—
Mind bender^b^	−0.21	.001	—	—	0.08		.41	—	—
Tap the emotion	—	—	−0.20	<.001	—		—	0.02	.83
Face to face^b^	—	—	−0.09	.10	—		—	0.01	.93
Emotional face	−0.04	.56	—	—	0.04		.74	—	—

^a^Nonparametric test provided given violation of normality.

^b^Raw task output was inverted so a larger output indicated better performance.

**Table 5:** Normative data by age bin for ages 17-50 years.

Assessments	Age bin (years)										
	17-20			21-30			31-40			41-50	
	Participant, n	Mean (SD)	Participant, n		Mean (SD)	Participant, n		Mean (SD)	Participant, n		Mean (SD)
Beep seeker	76	3.76 (4.06)	63		5.11 (4.57)	22		6.91 (5.87)	32		6.50 (4.94)
Sound sweeps	102	1.08 (0.29)	62		1.04 (0.32)	19		0.84 (0.33)	22		0.88 (0.27)
Bubble pop	94	5.59 (0.95)	68		5.41 (1.13)	23		5.04 (1.01)	33		4.93 (1.17)
Path finder	91	61.86 (21.22)	70		59.79 (25.02)	25		55.16 (24.87)	33		46.18 (21.99)
Mind bender	97	−8.7 (0.95)	56		−8.9 (0.94)	20		−8.82 (0.96)	23		−9.59 (0.84)
Tap the emotion	136	86.29 (10.91)	108		74.76 (20.27)	32		72.56 (21.05)	39		74.32 (18.31)
Face to face	154	−1.96 (0.41)	80		−1.95 (0.38)	22		−2.17 (0.35)	25		−2.09 (0.42)
Emotional face	62	56.42 (76.89)	63		53.82 (73.4)	24		67.65 (91.75)	32		36.49 (64.09)

**Table 6:** Normative data by age bin for ages 51-80 years.

Assessments	Age bin (years)							
	51-60			61-70			71-80	
	Participant, n	Mean (SD)	Participant, n		Mean (SD)	Participant, n		Mean (SD)
Beep seeker	35	6.29 (4.93)	34		3.97 (3.66)	7		3.71 (4.11)
Sound sweeps	28	0.89 (0.28)	29		0.79 (0.38)	7		0.7 (0.40)
Bubble pop	32	4.57 (1.08)	33		3.93 (0.99)	10		3.68 (0.92)
Path finder	49	45.96 (25.25)	45		34.98 (21.28)	10		35.7 (21.89)
Mind bender	28	−10.05 (1.07)	28		−9.62 (0.74)	4		−9.43 (0.48)
Tap the emotion	51	78.80 (14.93)	48		75.6 (18.52)	12		62.92 (25.71)
Face to face	30	−2.11 (0.39)	24		−2.33 (0.56)	4		−2.29 (0.25)
Emotional face	35	55.09 (73.18)	30		52.26 (107.65)	6		38.07 (110.4)

Most visual tasks were significantly associated with one another, with Spearman rho coefficients ranging from 0.19 to 0.42, indicating some expected shared variance and evidence for the measurement of unique constructs (Table 7). Beep seeker and bubble pop, which measure similar memory processes across auditory and visual domains, were also correlated with one another; however, the Spearman rho was only 0.28, suggesting that they largely capture distinct constructs. In addition, although significant, sound sweeps was found to be only minimally associated (ρ=0.16-0.36) with other theoretically distinct constructs (eg, visual-spatial learning and emotion detection).

**Table 7:** Online neurocognitive assessments’ correlation matrix.

Assessments		Beep seeker	Sound sweeps	Bubble pop	Path finder	Mind bender	Tap the emotion	Face to face
**Sound sweeps**								
	ρ^a^	N/A^b^	—^c^	—	—	—	—	—
	*P* value	N/A	—	—	—	—	—	—
**Bubble pop**								
	ρ	0.28	0.18	—	—	—	—	—
	*P* value	.002	.05	—	—	—	—	—
**Path finder**								
	ρ	0.1	0.36	N/A	—	—	—	—
	*P* value	.22	<.001	N/A	—	—	—	—
**Mind bender**								
	ρ	0.24	0.31	0.19	0.23	—	—	—
	*P* value	.05	<.001	.05	.005	—	—	—
**Tap the emotion**								
	ρ	0.02	0.16	0.22	0.25	0.42	—	—
	*P* value	.83	.04	.001	.006	<.001	—	—
**Face to face**								
	ρ	0.09	0.20	0.14	0.23	0.23	0.19	—
	*P* value	.43	.006	.15	.003	.001	.005	—
**Emotional face**								
	ρ	−0.21	N/A	−0.09	0.11	N/A	0.07	−0.05
	*P* value	.005	N/A	.35	.38	N/A	.32	.55

^a^ρ: Spearman's rank correlation coefficient.

^b^N/A: not applicable; the tasks were not included in the same battery.

^c^Redundant information.

## Discussion

### Principal Findings and Significance

In this study, we tested a new web-based neurocognitive assessment battery for individuals from the general community. Normative metrics were collected across 8 tasks measuring auditory discrimination, auditory perception, visual-spatial working memory, visual-spatial learning, cognitive flexibility, emotion detection, emotion identification, and inhibitory control of emotion.

Participants found the assessments to be widely acceptable across a range of ages. Age was negatively associated with the extent to which participants were able to understand task instructions; however, the broad majority of individuals in older age groups found the tasks understandable. Still, future iterations of these tasks should consider adaptations to better reach these individuals, such as improving task instructions, increasing practice trials, embedding video tutorials, or lowering the starting level of difficulty to reduce initial frustration. Despite finding the tasks widely interesting and enjoyable, fewer individuals indicated an interest in playing games at home. This difference may suggest that fewer participants would choose to engage with these tasks as a leisure activity but may be open to completing them if recommended by a member of their health care team. It is important to note that contrary to the survey wording, these tasks are not considered to be games and should not be interpreted as such. In contrast to traditional neuropsychological assessments, these measures are brief, allowing clinicians to collect a wealth of cognitive information in a relatively short period. These findings highlight the acceptability and efficiency of the battery.

Participant age was associated with 6 of the 8 tasks, with younger participants performing better than older participants. This trend has been widely observed in the cognitive literature [35-37]. Therefore, normative task data were stratified by age. Hearing sensitivity and sensitivity to mistuned, oddball, or discontinuous tones have been shown to deteriorate with age [38-40]. The slowing of processing speed with age has also been well documented [41,42]. Visuospatial working memory performance also decreases with age, potentially because of differences in chunking strategies and proactive interference [43-45]. Better performance observed with path finder, a task of visuospatial learning, may be due in part to more efficient within-task adaptability by younger individuals, a result identified previously with visuospatial tasks [43]. Other similar age-related associations have been documented in learning paradigms [46]. The findings of this study of decreased cognitive flexibility with age are also largely supported by other studies, such as those examining task-switching capacity [47,48]. Finally, in the literature, older age has been found to be related to poorer emotion recognition, processing facial emotion, and inhibitory control [49-51]. These results likely explain why performance on tap the emotion and face to face, tasks of emotion processing and inhibitory control, was negatively associated with age; however, it is unclear why emotional face did not show an association. Given these findings, which are supported by the greater cognitive literature, normative task data were stratified by age. Therefore, normative task data were stratified by age.

Better performance on a visual learning task was observed in men, corroborating previous gender findings with learning tasks that involve spatial navigation and manipulation [52,53]. These results appear to follow previous findings, suggesting male advantage in detecting interaural time and intensity differences, complex masking tasks, and lateralization of auditory discrimination processing, although these differences are small [54,55]. With samples larger than those in this study, age- and gender-specific norms could be constructed to better characterize the individual performance.

Although previous research has demonstrated associations between performance on cognition and education tests, our task battery did not show relationships with GPA or years of education after controlling for age [56,57]. These findings may indicate that the ONAs successfully captured neural system functioning as opposed to more notion-based intelligence. In addition, only bubble pop performance was related to the occupation level after controlling for age, suggesting a potential benefit of work status.

The majority of predominantly visual ONAs were only minimally associated with one another, as expected, suggesting that they are likely to tap into unique domains of performance. Beep seeker and bubble pop, which measure sensory memory and working memory across auditory and visuospatial domains, respectively, were related. Previous research has supported this association and provided evidence for similar neural mechanisms underlying both memory systems [58,59]. In addition, 2 of the 3 tasks of emotion processing were only minimally correlated with one another. This finding is expected given the varying demands on processes of inhibition between the tasks and differences in the facial targets (eg, responding to faces with any emotion vs identifying faces with matching emotions). It is surprising that tap the emotion and emotional face were not associated, given that both tasks involved processing of facial emotions while inhibiting a prepotent response, although it is unclear whether this relationship would exist with a larger sample who performed both tasks.

Despite the recent development of various digital cognitive assessment tools, few studies have released normative data sets, thus limiting the information that can be gained from these tasks. Collecting normative data from the general community is an important and necessary first step before interpreting cognitive performance in clinical populations. The current global health crisis has arrested the ability to administer traditional in-person cognitive assessments in clinical and research settings. The development of valid and reliable assessment platforms, which can be delivered remotely, has become crucial.

### Limitations and Future Work

Despite the strengths of our results, a few limitations exist that should be considered. Although 74.1% (579/781) of the sample came from the general community, a subset of participants was recruited through their attendance at a large public college institution. Therefore, young adults’ performance in this sample may differ somewhat from that of the general population. Due to the limited overlap of measures that each participant completed, we were unable to evaluate whether subsets of the tasks were explained by common underlying factors to investigate construct validity. Similarly, the correlations between tasks should be interpreted with caution, given the variability in the number of individuals who were administered each task. Data on participant race and ethnicity were also not collected as part of this study. In addition, 2 individuals identified as intersex prevented our ability to reliably predict how a greater population of intersex individuals may perform on the presented tasks. Future work should aim to evaluate race and gender minorities more specifically to better understand the performance and feasibility of these populations.

Experiential cognitive assessment in patients’ homes may help gain a better understanding of their true cognitive states, but it also raises the possibility that individuals will lack the attention or motivation to properly engage in testing [9]. Failure to prevent some patients from using substances or eliciting outside help during testing may also be a necessary aspect of remote testing [24,60]. In these situations, performance on a given task may not truly represent the intended cognitive domain, as it is likely to be in a distraction-limited environment. More information is also needed to understand how cognition in the general community fluctuates from shorter to more extended periods when assessed remotely.

The accessibility of these and other digital assessments provides an opportunity to integrate objective behavioral assessments directly into medical records to guide care. Given that the field of health informatics is still budding, future work should evaluate the extent to which these tasks capture unique variance and predict outcomes amidst other data.

### Conclusions

This study presented the performance of individuals from the general community using a novel cognitive assessment tool that can be employed remotely. Participants found the tasks to be interactive and easy to use. The development and validation of web-based tasks such as these widely expand the accessibility of cognitive assessment to new populations such as rural groups and others with limited physical mobility and may also increase the ability of health practitioners to conduct repeated testing. Quick, easy-to-use digital assessment platforms with remote capabilities such as this may help bring the field closer to achieving more impactful patient-centered care.

## Appendix

Multimedia Appendix 1 Supplementary material including online neurocognitive assessment task links and task distributions.

Multimedia Appendix 2 Participant usability feedback for online neurocognitive assessments.

## Abbreviations

GPA: grade point average
MSF: Minnesota State Fair
ONA: online neurocognitive assessment
RT: reaction time
UMN: University of Minnesota
