# Supplementary material for: Testing a Novel Web-Based Neurocognitive Battery in the General Community: Validation and Usability Study
Source: J Med Internet Res. 2021 May 6;23(5):e25082. doi: 10.2196/25082 (PMC8138705; doi:10.2196/25082)
Supplement: Multimedia Appendix 1 [file jmir_v23i5e25082_app1.docx]

# Supplementary Materials

## Online Neurocognitive Assessments (ONAs):

Register for a free account at [BrainHQ.com](file:///C:\Users\riley\Downloads\brainhq.com) and visit the following links to sample the online neurocognitive assessments presented in this study:

- Beep Seeker: [*https://assessment.brainhq.com/?#assessment/B2*](https://assessment.brainhq.com/?#assessment/B2)
- Sound Sweeps: <https://assessment.brainhq.com/?#assessment/1E>
- Bubble Pop: <https://assessment.brainhq.com/?#assessment/97>
- Pathfinder: <https://assessment.brainhq.com/?#assessment/A6>
- Mind Bender: <https://assessment.brainhq.com/?#assessment/0C>
- Tap the Emotion: <https://assessment.brainhq.com/?#assessment/9C>
- Face to Face: <https://assessment.brainhq.com/?#assessment/13>
- Emotional Face: <https://assessment.brainhq.com/?#assessment/9B>

## ONA Task Distributions


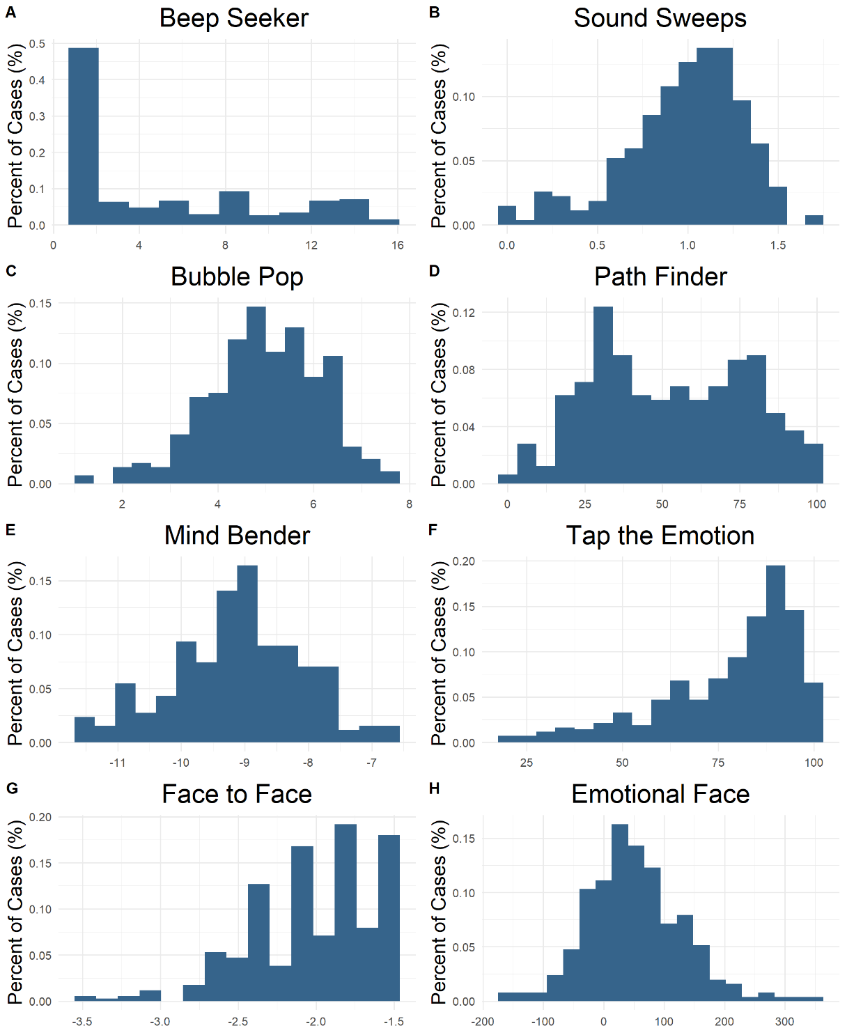


Supplementary Figure 1: Distributions of 8 online neurocognitive assessments
