# Supplementary figures and images for: Testing a Novel Web-Based Neurocognitive Battery in the General Community: Validation and Usability Study
Source: J Med Internet Res. 2021 May 6;23(5):e25082. doi: 10.2196/25082 (PMC8138705; doi:10.2196/25082)

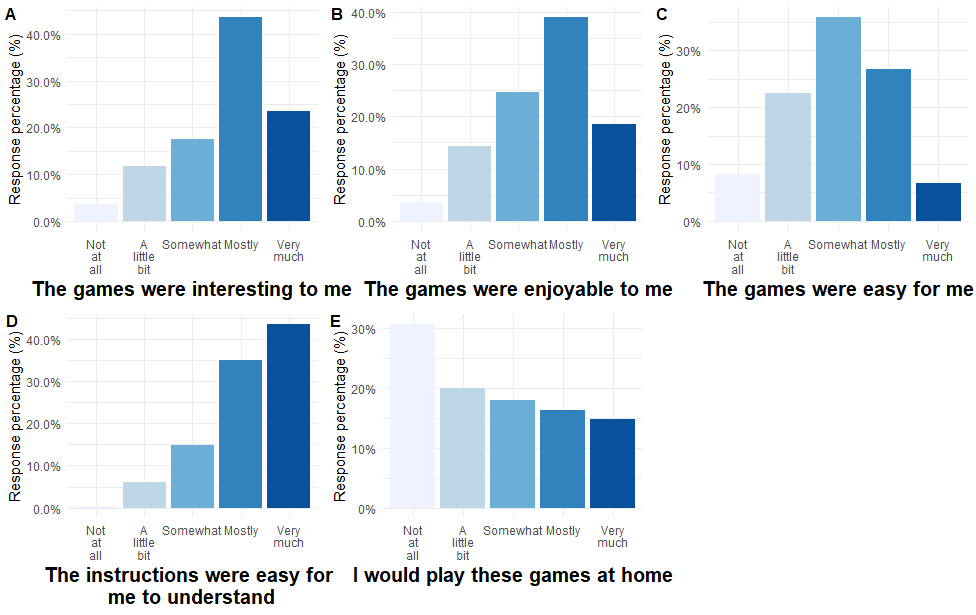

Supplement: Multimedia Appendix 2 [file jmir_v23i5e25082_app2.png]
